# Improvement in Turn-Off Loss of the Super Junction IGBT with Separated n-Buffer Layers

**DOI:** 10.3390/mi12111422

**Published:** 2021-11-19

**Authors:** Ki Yeong Kim, Joo Seok Noh, Tae Young Yoon, Jang Hyun Kim

**Affiliations:** School of Electrical Engineering, Pukyong National University, Busan 48513, Korea; kds9510@pukyong.ac.kr (K.Y.K.); shwntjr001@pukyong.ac.kr (J.S.N.); yoon1806@pukyong.ac.kr (T.Y.Y.)

**Keywords:** super junction, IGBT, p-pillar, n-buffer layer, on-state voltage, breakdown voltage, turn-off loss

## Abstract

In this study, we propose a super junction insulated-gate bipolar transistor (SJBT) with separated n-buffer layers to solve a relatively long time for carrier annihilation during turn-off. This proposition improves the turn-off characteristic while maintaining similar on-state characteristics and breakdown voltage. The electrical characteristics of the devices were simulated by using the Synopsys Sentaurus technology computer-aided design (TCAD) simulation tool, and we compared the conventional SJBT with SJBT with separated n-buffer layers. The simulation tool result shows that turn-off loss (E_off_) drops by about 7% when on-state voltage (*V*_on_) and breakdown voltage (BV) are similar. *V*_on_ increases by about 0.5% and BV decreases by only about 0.8%.

## 1. Introduction

The power semiconductor is a switching device characterized by high voltage and high current. It is generally composed of a bipolar junction transistor (BJT), insulated-gate bipolar transistor (IGBT), power metal-oxide-semiconductor field-effect transistor (power MOSFET) and thyristor [1,2,3,4]. These devices are used in a wide range of fields including high-power three-phase motor control inverters as well as a boost converter of UPS and industrial equipment power supplies. Recently, as the electric vehicle market is increasing, the importance of power semiconductors is growing [5,6,7]. Among the power semiconductors, a super junction IGBT (SJBT) [8,9,10] has been gaining popularity due to an important component of high-efficiency power conversion systems. Compared to a power MOSFET, the SJBT has low resistance values because it has a p-doped layer (p-collector) at the anode side which injects holes to the drift region [11,12,13]. In addition, the SJBT has an n-pillar and a p-pillar which has opposing doping to make charge compensation, resulting in full depletion of both pillars. As a result, the deformed electric field distribution helps to make the device thinner while maintaining breakdown voltage (BV) characteristics. In a previous study, the operation of SJBT was divided into unipolar mode and bipolar mode according to the current flow in both pillars [8,9,10]. First, there is a unipolar mode in which each pillar has a full reverse bias and a bipolar mode where some reverse bias is removed. At this time, the operation trigger between the two modes is defined as an n-buffer layer doping concentration. As the doping concentration of the n-buffer layer decreases, the barrier of the PNP transistor, composed of the p-collector layer, the n-buffer layer and the p-pillar, lowers and the injection of holes increases. As a result, the reverse bias between the p-pillar and the n-pillar is removed, and the number of minority carriers, as well as the majority of each pillar, increases by diffusion [14]. Therefore, the resistance in the pillar is reduced by conductivity modulation [15], and the on-state voltage (*V*_on_) decreases. However, the increased minority carriers in each pillar induce a tail current [16,17], which cannot be extracted quickly at the off-state. Then it deteriorates the turn-off loss (E_off_). Therefore, in the SJBT, there is a trade-off between *V*_on_–E_off_ according to the doping concentration of the n-buffer layer change, and this needs to be improved [18,19,20,21].

This paper aimed to improve the off-state characteristics by varying the concentration of the n-buffer layer in contact with each pillar [22]. We compared the on-state characteristics (3.1), BV characteristics (3.2), and turn-off characteristics (3.3) between the conventional SJBT (C-SJBT) and the SJBT with separated n-buffer layers (SB-SJBT). We verified each characteristic and finally explained the improvement of the trade-off (3.4).

## 2. Materials and Methods

### 2.1. Structure of the Proposed IGBT with Separated n-buffer Layers

**Figure 1:**
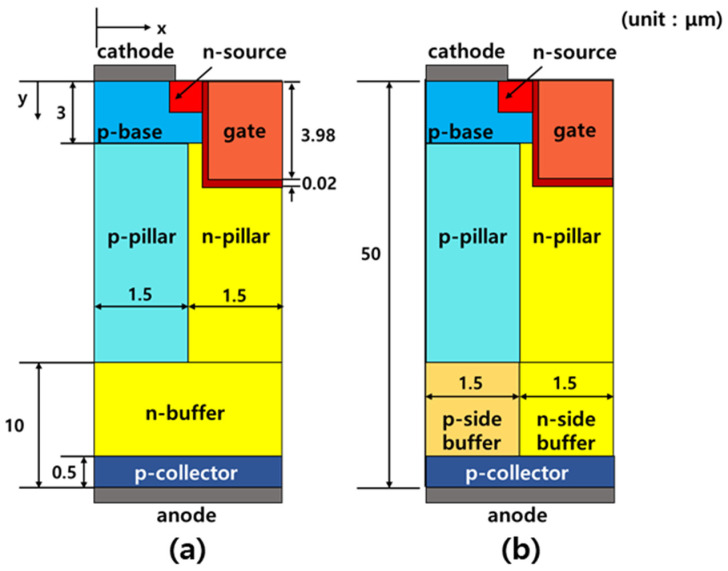
Structure of (**a**) C-SJBT and (**b**) proposed SJBT with separated n-buffer layers.

The structure of C-SJBT and proposed super junction IGBT with SB-SJBT are illustrated in Figure 1. In order to identify the characteristic difference between C-SJBT and SB-SJBT, we have simulated the electrical characteristics of devices by using the Synopsys Sentaurus technology computer-aided design (TCAD) simulation tool. In the simulation, we designed structures with the parameters in Table 1. The parameters of SB-SJBT were very similar to C-SJBT. The only difference between C-SJBT and SB-SJBT was the existence of the separated n-buffer layer. It was composed of a p/n-side n-buffer layer that was in contact with each p/n-pillar.

### 2.2. Applied Model Physics in Simulation

To accurately analyze the electrical and thermal characteristics, band-gap narrowing (BGN), thermodynamic and analytical expressions to calculate the thermoelectric power (AnalyticTEP) were used as physics by the Synopsys Sentaurus TCAD simulation tool. Moreover, inversion and accumulation layer mobility (IALMob) and high-field saturation were used as mobility models, and Shockley–Read–Hall (SRH), Auger electron spectroscopy (AES) and avalanche generation (Lackner) were used as recombination models [23].

## 3. Results and Discussion

### 3.1. Basic Characteristics of SJ-IGBT

**Figure 2:**
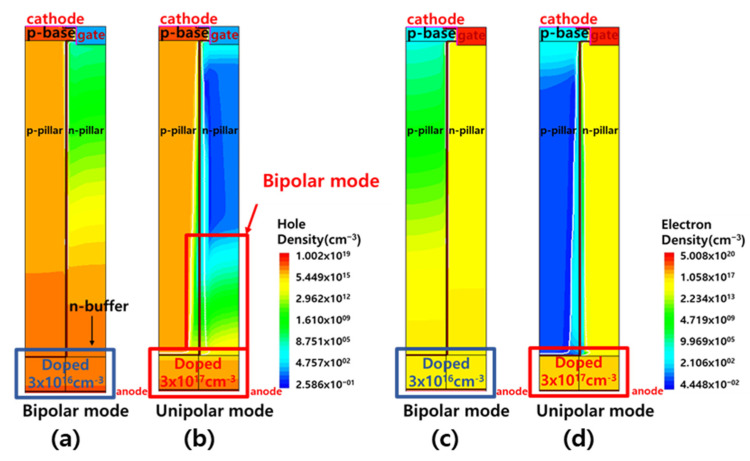
Hole density of (**a**) bipolar mode and (**b**) unipolar mode and electron density of (**c**) bipolar mode and (**d**) unipolar mode. The doping concentrations of the n-buffer layer are $3\times 10^{16}$ cm^−3^ and $3\times 10^{17}\;$ cm^−3^ in bipolar and unipolar mode respectively. In Figure 1b, the hole is diffused to the n-drift region and is a minority carrier which reduces turn-off characteristics.

As previously described, the SJBT has a unipolar mode and a bipolar mode, which vary depending on the doping concentration of the buffer. To verify whether the two modes operate, hole density and electron density of structures were calculated through two structure simulations that differed only in doping concentrations. In Figure 2, there is a structure with buffer doped $3\times 10^{16}$ cm^−3^ at Figure 2a,c, and a structure with buffer doped $3\times 10^{17}\;$ cm^−3^ at Figure 2b,d. In Figure 2a,c, the structure with a lightly doped buffer was set to bipolar mode, which made more carriers pass through the buffer, increasing the diffusion between the pillars. Conversely, in the case of a buffer with a higher concentration as shown in Figure 2b,d, the structure was set to unipolar mode, which suppressed the hole injection out of the p-pillar and reduced the absolute number of carriers in the p-pillar. According to the simulation results, Figure 2d shows that the unipolar mode operated in the electron density as expected, but in Figure 2b, it is observed that a part of the anode operated in bipolar mode, as opposed to what we expected to operate in unipolar mode. We attribute the reason to the fact that the carriers passing through the buffer were holes, and going from p-pillar to n-pillar was easier than going from n-pillar to p-pillar. The difference in mobility between the hole and electron is another reason [24].

### 3.2. The Way to Compare Characteristics

**Figure 3:**
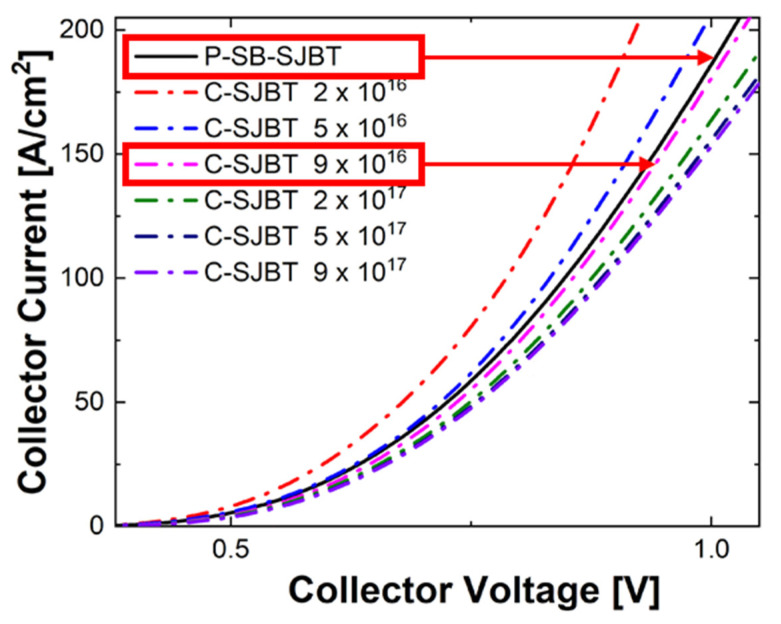
*I*_c_-*V*_c_ characteristic graph of SB-SJBT and C-SJBT. The standard of measuring *V*_on_ is the voltage corresponding to current 100 A/cm^2^.

We propose a method to improve the off-state characteristics by controlling holes through partially regulating the concentration of the n-buffer layer in IGBT. To compare conventional IGBT and the proposed IGBT thoroughly, we set the target doping concentration in the n-buffer layer with the following two conditions. First, referring to Figure 1a, in the bipolar mode, the hole injected into the p-pillar is diffused in the entire n-pillar region. Because the role of the separated buffer is to control holes in a limited area (p-pillar), the influence of the separated buffer is reduced in the bipolar mode. Therefore, the structure needs to operate in a more distinct unipolar mode. Second, the doping concentration of the n-buffer layer affects the on-state conductivity, which can be evaluated through the *V*_on_ parameter in output characteristics. If the buffer concentration is too high, the structure clearly operates in a unipolar mode, as the *V*_on_ characteristic deteriorates, and if the buffer concentration is too low, it operates in a bipolar mode. Therefore, by controlling the doping concentration, we set the target doping concentration considering the similarity of *V*_on_ and unipolar operation. Because of this relationship, we set the buffer concentration of SB-SJBT to $3\times 10^{17}$ cm^−3^ in the p-side and $3\times 10^{16}$ cm^−3^ in the n-side. The *I*_c_-*V*_c_ characteristic curve of this SB-SJBT is plotted in Figure 3, and the characteristic curve of the C-SJBT according to the buffer concentration is also plotted in order to specify the concentration of C-SJBT with similar *V*_on_ characteristics. According to Figure 3, C-SJBT, which has the closest *V*_on_ characteristic to that of SB-SJBT, has a buffer concentration of $9\times 10^{16}\;$ cm^−3^. Additionally, in order to investigate the effect of partially changing doping concentration, a structure in which the doping concentration of both sides is exchanged is also used as a comparison object. The first proposed structure is defined as P-SB-SJBT because the concentration of the p-side is higher than that of the n-side. Furthermore, a structure in which both concentrations are reversed is defined as N-SB-SJBT. Parameters are arranged in Table 2. In addition, to confirm variable characteristics such as trade-off characteristics between E_off_ and *V*_on_, the properties were compared according to the concentration of the p-collector at the same temperature. The default value of the p-collector concentration is 10^18^ cm^−3^ and increases from $3\times 10^{17}$ cm^−3^ to $2.4\times 10^{18}\;$ cm^−3^. There is no reason to separate the buffer if the concentration of the p-collector is less than 10^17^ cm^−3^ since the number of holes entering the n-drift is small. As such, the higher the concentration, the greater the effect of SB-SJBT. However, if the concentration is more than 10^19^ cm^−3^, the number of minority carriers staying in the n-drift region becomes excessively large and the E_off_ increases [25,26,27]. Similarly, if the concentration of the n-buffer is too low, the number of holes passing through and entering the n-drift increases, resulting in bad E_off_ characteristics [18,19].

### 3.3. On-State Characteristics

**Figure 4:**
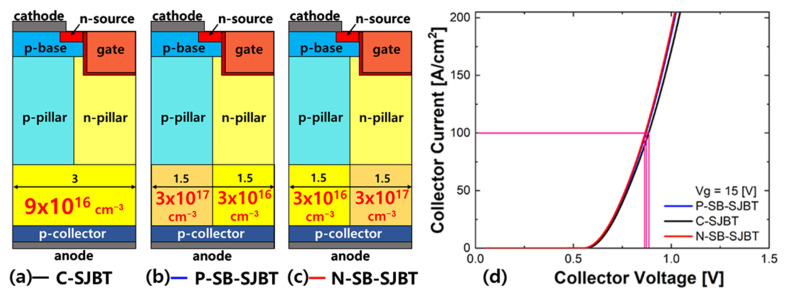
(**a**) C-SJBT in which the buffer is uniformly doped, (**b**) P-SB-SJBT in which the buffer below the p-pillar side is doped higher than the buffer below the n-pillar side and (**c**) N-SB-SJBT in which the concentration of each buffer is switched in the structure of P-SB-SJBT. (**d**) On-state *I*_c_-*V*_c_ characteristic curves of C-SJBT, P-SB-SJBT, and N-SB-SJBT. (*V*_g_ = 15 V).

**Figure 5:**
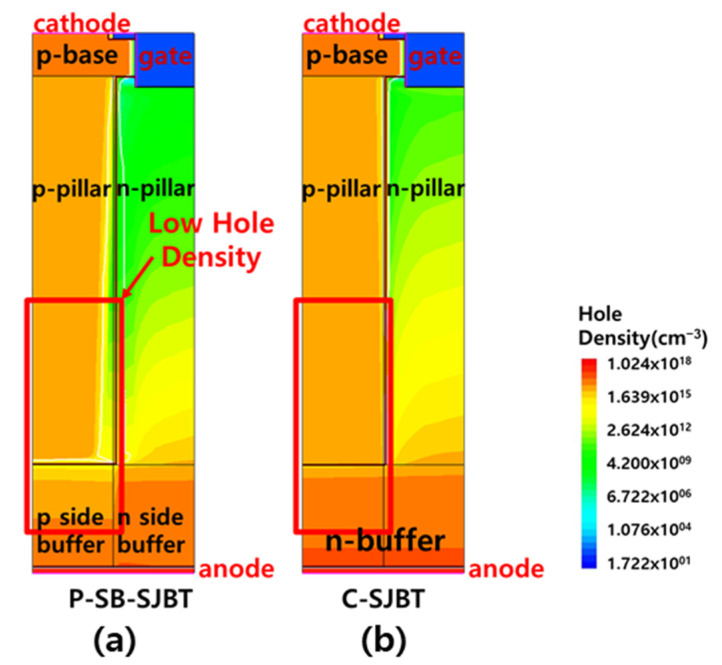
(**a**) Hole density of P-SB-SJBT, and (**b**) C-SJBT. Buffer concentration of (**a**) consist of $3\times 10^{17}$ cm^−3^ and $3\times 10^{16}$ cm^−3^, and concentration of (**b**) consist of only $3\times 10^{16}$ cm^−3^.

Figure 4 shows the structure and on-state *I*_c_-*V*_c_ characteristic curves of C-SJBT, P-SB-SJBT and N-SB-SJBT. It shows that the on-state characteristic of the P-SB-SJBT is slightly higher than that of the C-SJBT. When the gate voltage is applied at 15 V and collector current is 100 (A/cm^2^), the on-state voltages of C-SJBT, P-SB-SJBT and N-SB-SJBT are respectively 0.87 V, 0.86 V and 0.85 V. The on-state voltage of P-SB-SJBT is about 1% lower than conventional super junction IGBT. Generally, in the P-SB-SJBT, the presence of the p-side n-buffer layer becomes a barrier preventing hole injection in the p-collector layer. The p-side n-buffer layer, which is relatively higher than the n-side n-buffer layer, increases the recombination rate of holes and consequently reduces the holes reaching the p-pillar. As a result, the absolute number of carriers present in the p-pillar is reduced, which causes lower on-state characteristics than conventional SJBT. The difference of hole injection between SB-SJBT and C-SJBT is shown in Figure 5. Under the same conditions, except concentration of the n-buffer, fewer holes enter through a highly doped buffer (Figure 5a). The lower the carriers, the lower the *V*_on_. However, as shown in Figure 4, because this paper set *V*_on_ similar to P-SB-SJBT and C-SJBT, there is no large error.

### 3.4. Breakdown Characteristics

**Figure 6:**
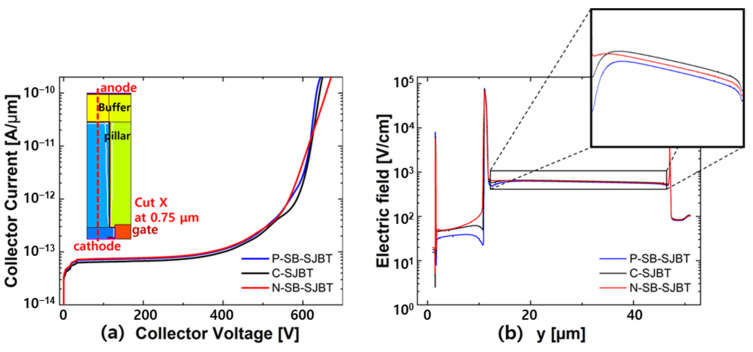
(**a**) The breakdown voltage characteristic and (**b**) electric field distribution in the p-pillar (x = 0.75 µm).

Figure 6a,b shows the breakdown voltage characteristics and electric field distribution in the p-pillar. From Figure 6a, we can see that the breakdown voltage of P-SB-SJBT is 632.7 V, which decreased by 0.51% compared to C-SJBT of 635.9 V. It also increased by 0.72% compared to N-SB-SJBT of 627.5 V. The difference between the breakdown voltage of the two devices appears as a change in the slope of the electric field distribution due to the difference in doping concentration of the p-side n-buffer layer. Since the width of the structure is 3.0 µm, the electric field distribution was measured according to y (=length) at the p-pillar near x (=width) of 0.75 µm which is half of the p-pillar. The result is shown in Figure 6b. Because the structure consists of a super junction, the electric field is flat between 12 µm and 47 µm of the structure length, where the p-pillar is located. In the enlarged figure, we can see that the electric field is formed in the order of C-SJBT, N-SB-SJBT, and P-SB-SJBT. BV tends to be in the order described above, but fluctuations of less than about 0.5% are negligible [28].

### 3.5. Turn-Off Characteristics

#### 3.5.1. The Way to Calculate Turn-Off Loss

**Figure 7:**
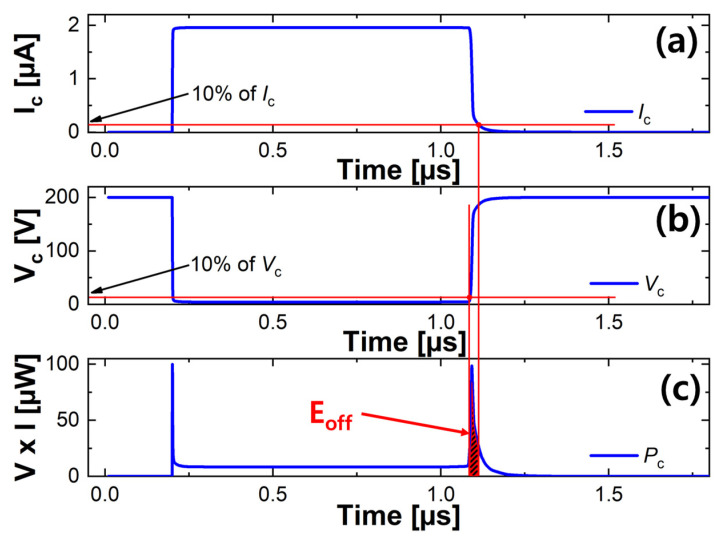
The way to calculate the turn-off loss: (**a**) the turn-off waveform of collector current; (**b**) collector current; and (**c**) power dissipation. Red lines are drawn vertically at times corresponding to 10 percent of collector total current and 10 percent of collector voltage.

We calculate the E_off_ in the following ways to verify the turn-off characteristics. Figure 7 shows an example of a characteristic curve of the C-SJBT in which the buffer is doped with $9\times 10^{16}\;$ cm^−3^ to calculate E_off_. There is collector current (*I*_c_) as in Figure 7a, collector voltage (*V_c_*) as in Figure 7b, and power dissipation (*P* = *V*_c_ × *I*_c_) as in Figure 7c. E_off_ was defined as the integral of the product of voltage and current (*P*) from the time corresponding to 10% of the current to the time corresponding to 10% of the voltage. The diagonally filled area in Figure 7c is the turn-off loss [29].

#### 3.5.2. E_off_ on P-SB-SJBT and C-SJBT

**Figure 8:**
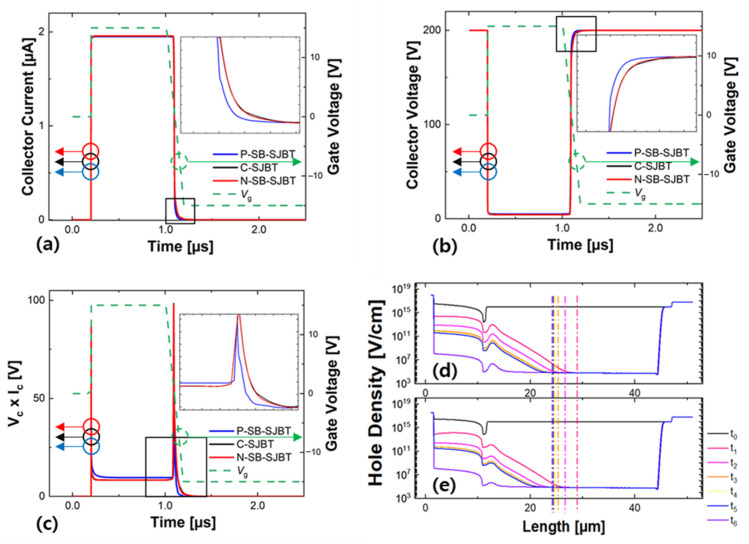
(**a**) The way to calculate the turn-off loss: the turn off waveform of collector current; (**b**) collector voltage; and (**c**) power dissipation. The rest are hole density in p-pillar of (**d**) C-SJBT and (**e**) P-SB-SJBT respectively.

In Figure 8, the gate voltage that changes from 0 V → 15 V → −15 V according to short time is applied to C-SJBT and SB-SJBT. It can be seen, from the waveform of collector current (Figure 8a) and collector voltage (Figure 8b), that the turn-off speed of the proposed P-SB-SJBT is faster than that of C-SJBT. The enlarged graph on the upper right shows the section with a change in detail. The p-side n-buffer layer with a high doping concentration helps the hole in the p-pillar to be extracted quickly. In addition, the reduced number of carriers in the on-state characteristic improves the turn-off characteristic. Figure 8d,e shows the process of extracting in the p-pillar during turn-off with hole density. Depending on the time the hole is extracted, *t*0 to *t*6 are indicated, which are 0.7 × 10^−6^ s, 0.9 × 10^−6^ s, 1.2 × 10^−6^ s, 1.4 × 10^−6^ s, 1.7 × 10^−6^ s, 1.8 × 10^−6^ s, 1.9 × 10^−6^ s, and 2.0 × 10^−6^ s, respectively. A vertical line is drawn from the time when the holes in C-SJBT are completely extracted. When the extended vertical line is connected with the time in Figure 8e, the corresponding time is slower than the time corresponding to completely extracted holes from P-SB-SJBT. Therefore, P-SB-SJBT (Figure 8e) extracts the holes in the p-pillar faster than the C-SJBT (Figure 8d) for the same time. As a result of calculating E_off_ in the manner described in the previous paragraph, P-SB-SJBT, C-SJBT, and N-SB-SJBT have losses of 1.28 µJ, 1.39 µJ, 1.44 µJ, respectively. The turn-off characteristic of P-SB-SJBT is advanced by about 7.45%, and 10.75% compared with C-SJBT and N-SB-SJBT.

### 3.6. Trade-Off and Characteristics Trend

**Figure 9:**
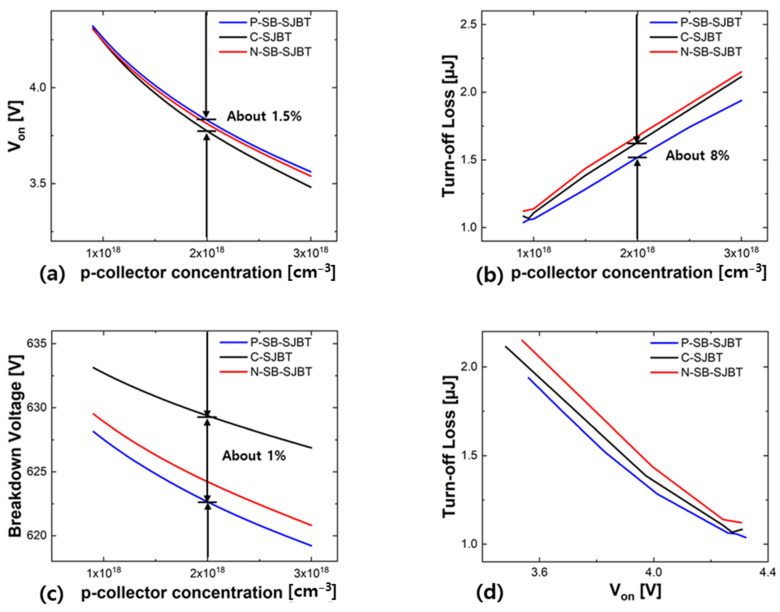
Result characteristics in this study. Even if the doping concentration of the p-collector is set to $2\times 10^{18}\;\;$ cm^−3^, the increase in (**a**) *V*_on_ is only about 1.5%, while the decrease in (**c**) BV is only about 1% but decrease in (**b**) E_off_ is about 8%. The proposed structure (P-SB-SJBT) has the advantage of E_off_ with similar *V*_on_ and BV. (**d**) This is consistent with the excellent trade-off characteristics of the proposed structure. Simulation is measured under the same conditions, such as temperature (300 K).

To observe the structure from various viewpoints, simulation was performed with the p-collector as a variable. The result is shown in Figure 9. In Figure 9a,c, characteristic curves of *V*_on_, E_off_, and BV with respect to the p-collector concentration are shown in order. The results, that each value is proportional to the concentration of the p-collector and the correlations between structures do not intersect, indicate that the P-SB-SJBT maintains a constant improvement over the C-SJBT regardless of other variables. Figure 9d shows the trade-off relationship between E_off_ and *V*_on_ of structures. In IGBT, a trade-off exists between E_off_ and *V*_on_, and among many evaluation methods for IGBT; the trade-off is generally used as an evaluation index. In the trade-off, E_off_ tends to decrease as *V*_on_ increases, and the closer the curve is to the origin, the better the structure is evaluated. Since the decrease in E_off_ is around 8% compared to the increase of around 1% in *V*_on_, it is natural that the trade-off improves by reflecting this. In Figure 9d, the P-SB-SJBT is much closer than C-SJBT. Therefore, P-SB-SJBT has a more improved performance than C-SJBT.

## 4. Conclusions

As one of the methods to solve the turn-off loss problem, this paper presents a method to control the absolute number of holes entering the p-pillar. It also accelerates the recombination of the remaining holes. Increasing the concentration of the overall buffer significantly reduces the number of flow holes, but when using a separated buffer to partially control the flow of holes, the BV decreases very slightly (0.51% reduction) compared to C-SJBT with similar *V*_on_, and the relative E_off_ characteristic improves greatly (7.45% reduction). Therefore, the separated buffer is effective in enhancing output characteristics. Improvement of this E_off_ would be useful because it is the biggest problem of SJBT, but since the concentration of the buffer is divided into two, it will need optimization to determine which combination is better to use.

## Figures and Tables

**Table 1:** Device parameters for the simulations.

Parameter	C-SJBT	SB-SJBT
width	3.0 µm	3.0 µm
length	50.0 µm	50.0 µm
gate depth	5.0 µm	5.0 µm
p/n-pillar width	1.5 µm	1.5 µm
p/n-side n-buffer width	-	1.5 µm
n-buffer layer depth	9.5 µm	9.5 µm
p-base doping	$6\times 10^{16}\;$ cm^−3^	$6\times 10^{16}\;$ cm^−3^
n-source doping	$5\times 10^{20}\;$ cm^−3^	$5\times 10^{20}\;$ cm^−3^
p/n-pillar doping	$1\times 10^{16}$ cm^−3^	$1\times 10^{16}\;$ cm^−3^
n-buffer doping	$9\times 10^{16}\;$ cm^−3^	-
p-collector doping	$1\times 10^{18}\;$ cm^−3^	$1\times 10^{18}\;$ cm^−3^

**Table 2:** Buffer doping concentration according to each structure.

Parameter	P-SB-SJBT	C-SJBT	N-SB-SJBT
p-side buffer doping	$3\times 10^{17}$ cm^−3^	$9\times 10^{16}$ cm^−3^	$3\times 10^{16}$ cm^−3^
n-side buffer doping	$3\times 10^{16}$ cm^−3^	$9\times 10^{16}$ cm^−3^	$3\times 10^{17}$ cm^−3^

## Data Availability

This study reported a poster presented at Nano Korea 2021 and it was cited at reference [22].
